# Selective targeting of histone modification fails to prevent graft versus host disease after hematopoietic cell transplantation

**DOI:** 10.1371/journal.pone.0207609

**Published:** 2018-11-19

**Authors:** Bader Alahmari, Matthew Cooper, Edward Ziga, Julie Ritchey, John F. DiPersio, Jaebok Choi

**Affiliations:** 1 Department of Medicine, Division of Oncology, Washington University School of Medicine, St. Louis, Missouri, United States of America; 2 Department of Pediatrics, Division of Hematology/Oncology, Washington University School of Medicine, St. Louis, Missouri, United States of America; Beth Israel Deaconess Medical Center, UNITED STATES

## Abstract

Allogeneic hematopoietic cell transplantation is often complicated by graft versus host disease (GvHD), primarily mediated through allo-reactive donor T cells in the donor stem cell graft. Enhancer of Zeste Homolog 2 (EZH2), a histone-lysine N-methyltransferase and a component of the Polycomb Repressive Complex 2, has been shown to play a role in GvHD pathology. Although not yet clear, one proposed mechanism is through selective tri-methylation of lysine 27 in histone 3 (H3K27me3) that marks the promoter region of multiple pro-apoptotic genes, leading to repression of these genes in allo-reactive T cells. We found that selective pharmacologic inhibition of H3K27me3 with EPZ6438 or GSK126 did not prevent murine GvHD. This suggests the GvHD mitigating properties of DZNep are independent from H3K27me3 inhibition. Furthermore, while pharmacologic inhibition of EZH2 by DZNep has been shown to be effective in abrogating mouse GvHD, we found that DZNep was not effective in preventing GvHD in a human T cell xenograft mouse model. Although EZH2 is an attractive target to harness donor allo-reactive T cells in the post-transplant setting to modulate GvHD and the anti-leukemia effect, our results suggest that more selective and effective ways to inhibit EZH2 in human T cells are required.

## Introduction

Allogeneic hematopoietic cell transplantation (Allo-HCT) remains the only curative therapy for relapsed and refractory hematologic malignancies. T cells in the donor graft mediate a beneficial graft versus leukemia effect (GvL), inducing remission and long term relapse free survival[1]. However, these same T cells induce life-changing and often life-threatening graft versus host disease (GvHD), characterized by skin, gastrointestinal tract and liver involvement[2]. Therapies mitigating GvHD while maintaining GvL remain elusive. Epigenetic regulation through DNA methylation and histone modification plays an important role in the expression and maintenance of T cell lineage-specific transcription factor genes[3]. We recently showed that epigenetic interventions using azacitidine, a DNA methyltransferase 1 (DNMT1) inhibitor, mitigates GvHD and preserves the GvL effect after murine allo-HCT through the in vivo induction of regulatory T cells[4] and the selective inhibition of donor effector T cell proliferation compared to donor regulatory T cells in vivo[5]. Polycomb Repressive Complex 2 (PRC2) is a transcriptional repressor that functions to silence the expression of developmental and differentiation genes in human cells through the tri-methylation of lysine 27 in histone H3 (H3K27me3) using its enzymatic subunit, the Enhancer of Zeste Homolog 2 (EZH2), which is a histone-lysine N-methyltransferase[6]. Human EZH2 shares 98% similarity with mouse homologue In normal physiological processes, EZH2 is expressed in actively dividing but not resting T cells[7]. EZH2 has an inhibitory role in T cell differentiation through H3K27me3 enrichment at T cell signature-cytokine loci, for example, *Ifng* locus in T helper (Th) 2 and *Il4* locus in Th1 [8]. Furthermore, EZH2 can maintain effector T cell survival through the suppression of multiple death receptor pathways[9]. Genetic deletion of *Ezh2* in donor T cells has demonstrated remarkable prevention of GvHD in murine allo-HCT models[10]. Furthermore, pharmacologic inhibition of EZH2 using DZNep, a non-specific histone methyltransferase inhibitor, resulted in significant abrogation of mouse GvHD with the preservation of GvL, likely through the induction of pro-apoptotic gene *Bim*[11]. However, the global histone methyltransferase inhibitory effect of DZNep on active and repressive histone marks could induce potential off target effects. Thus, we tested if selective pharmacologic inhibition of H3K27me3 using small molecule inhibitors EPZ6438 or GSK126 behaves similarly to DZNep in GvHD preclinical models, aiming to explore effective interventions with limited off target effects. In addition, it is difficult to translate the GvHD protection observed in murine allograft models into clinical studies without testing the effect of DZNep (or related compounds) in human T cells in vitro and in xenogeneic models of GvHD. Here we report that human and mouse T cells exhibit differential responses to DZNep, with DNZep treatment failing to mitigate GvHD in xenogeneic models of allo-HCT.

## Materials and methods

### Mice

C57BL/6J mice (CD45.1 or CD45.2), Balb/c mice and NOD/SCID/γc (NSG) mice were purchased from Jackson Laboratory (Bar Harbor, ME). Animal study protocols including animal care and euthanasia were approved by the Washington University School of Medicine Animal Studies Committee (Approval number 20150028).

### Allo-HCT and assessment of GvHD

Major histocompatibility complex (MHC) mismatched HCT (B6 → Balb/c) were performed as previously described[4, 12]. In brief, splenic pan T cells were isolated from B6 mice (H-2b, CD45.2+) and T cell-depleted bone marrow cells (TCD BM) isolated from congenic B6 mice (H-2b, CD45.1+). TCD BM (5x10^6^) and splenic pan T cells (5x10^5^) were transplanted (day 0) into lethally irradiated (925 cGy, day -1) allogeneic Balb/c recipient mice (H-2d, CD45.2+). For xenograft model, NSG mice were sub-lethally irradiated (250 cGy) at day -1. Frozen de-identified human peripheral blood mononuclear cells (PBMC) were collected in compliance with the protocols outlined by the Washington University School of Medicine Human Studies Committee, and as determined by Washington University’s Human Research Protection Office in 2009, these anonymous human blood cells are not considered human subjects research. Pan T cells were isolated from PBMCs using Miltenyi Human pan T isolation kit and purified using the AutoMACS (Miltenyi Biotech, Aubum, CA). Purified human T cells (3x10^6^) were injected into NSG mice at day 0 as described previously[13]. In all experiments, mice were maintained under pathogens-free conditions. Mice survival was monitored daily. Clinical GvHD score was assessed weekly as described previously [14]. Animals losing more than 20% of their starting body weights were euthanized.

### In vitro T cell activation

Murine T cells were isolated from B6 mouse spleen using mouse pan T isolation kit and purified using the AutoMACS (Miltenyi Biotech). Cells were plated in 24 well plates at a concentration of 3x10^5^/mL and activated for three days with anti-CD3/CD28 antibody-coated beads (Invitrogen, Carlsbad, CA) in 1:1 bead/cell ratio in 2 ml of Xcyte media supplemented with IL-2 (10 IU/mL)[4]. Human pan T cells were isolated from frozen human PBMCs, using human Pan T cell isolation kit (Miltenyi Biotech) using the AutoMACS (Miltenyi Biotech). Pan T cells were plated in 96 well plate at concentration of (1x10^5^/200uL) and activated for three days with anti-CD3/CD28 antibody-coated beads (Invitrogen, Carlsbad, CA) at a bead:cell ratio of 1:1 in OpTmizer medium (Invitrogen) supplemented with 5% Fetal Calf Serum, 1% L-glutamine, 1% penicillin/ Streptomycin and IL-2 (50 IU/ml).

### Flow cytometric analysis

The following antibodies were used for flow cytometric assays: For mouse T cells H3K27me3 (clone: mAbcam6147) (Abcam Inc. Cambridge, MA), CD8 (clone: 53–6.7) Invitrogen, Carlsbad, CA), CD4 (clone: GK1.5) (eBioscience). For human T cells, CD4 (clone: RPA-T4) and CD8 (clone: RPA-T8) (BD Biosciences). For both human and mouse T cells, anti-EZH2 (clone: 11/EZH2) (BD Biosciences) and Annexin V detection kit (eBioscience). For chimerism testing, mouse H2Kd (clone: SF1-1.1) and CD45.2 (clone: Ly5.2, LCA) were used (BD Biosciences). All data were collected on a FACScan cytometer (BD Biosciences, Mountain View, CA) and analyzed using FlowJo 9 (Tree Star Inc, Ashland, OR).

### EZH2 inhibitors

3-Deazaneplanocin A (DZNep) was purchased from Cayman chemical (Ann Arbor, MI). EPZ6438 and GSK126 were purchased from Xcessbio (San Diego, CA). All compounds were dissolved in 10% dimethyl sulfoxide (DMSO) in phosphate-buffered saline (PBS).

### Statistical analysis

Unpaired t-test was used to compare between two groups. Log-rank test was used to compare between curves. Multiple t-tests were used to compare between groups at multiple time points. One-way analysis of variance (ANOVA) was used to compare between multiple groups with Dunnett’s test to adjust for multiple comparisons. GraphPad Prism 7 (GraphPad Software Inc, La Jolla, CA) software was used for statistical calculation.

## Results

### Pharmacologic inhibition of EZH2 with DZNep prevents GvHD in murine allo-HCT

**Fig 1:**
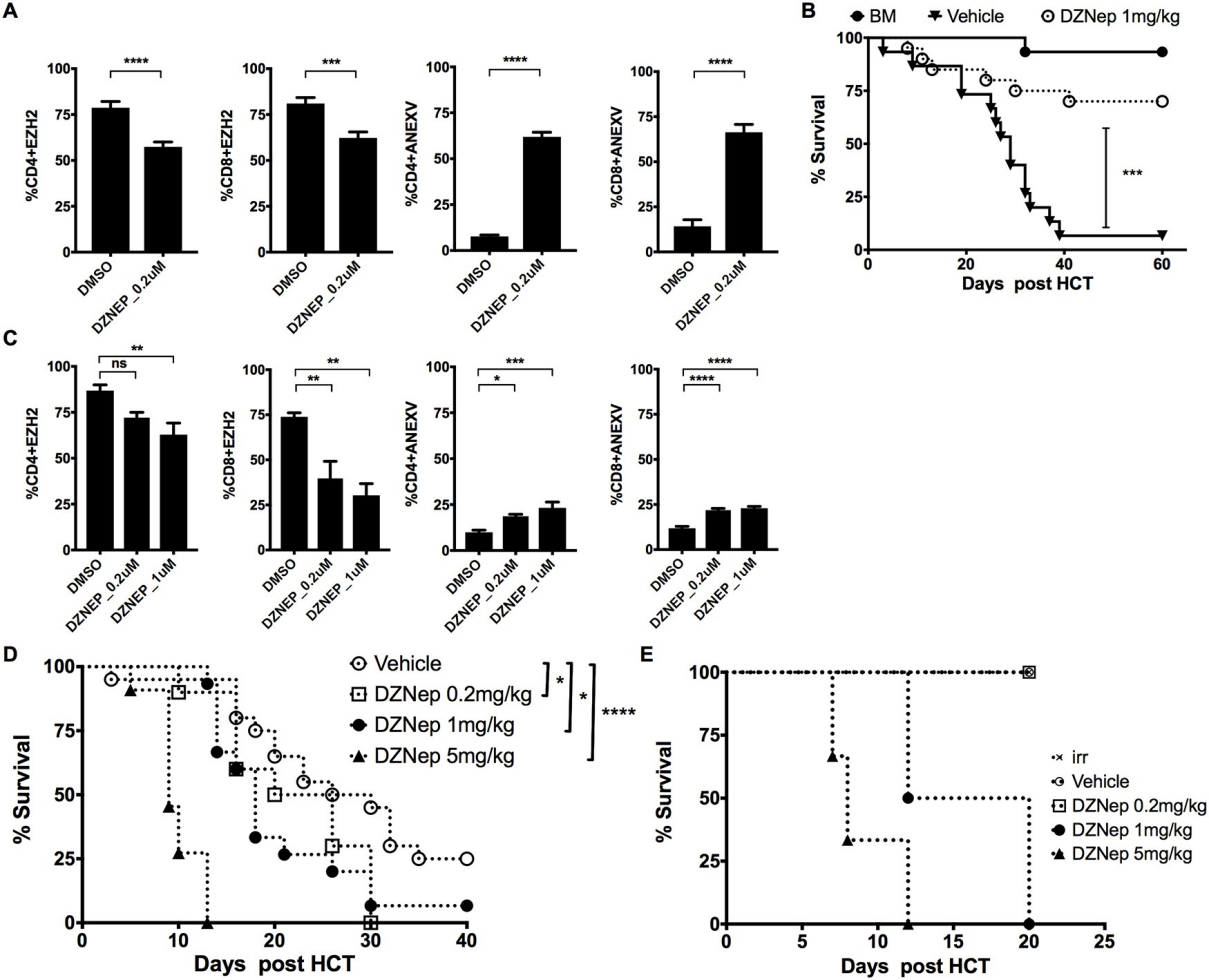
DZNep treatment is ineffective at mitigating GvHD in xenograft model. (A) Pan T cells were isolated from B6 mouse spleen and treated at day 0 with DZNep or 10% DMSO followed by activation with anti-CD3/CD28 antibody-coated beads for 3 days. T cells were then harvested, followed by intracellular staining for EZH2 or staining using Annexin V detection kit for flow cytometry. Data summary as mean ± SEM, pooled from 4 independent experiments. (B) MHC mismatched HCT (B6 → Balb/c) was performed as described in methods, vehicle (10% DMSO) or DZNep was injected every other day at 1mg/kg subcutaneous (s.c.) starting at day 3 post-transplant for a total of 8 doses, showing the survival graph for 15–20 mice each group pooled from 4 independent experiments. (C) Human pan T cells isolated from PBMCs and treated at day 0 with DZNep or 10% DMSO followed by activation with anti-CD3/CD28 antibody-coated beads for 3 days, T cells then harvested and followed by intracellular staining for EZH2 or staining using Annexin V detection kit for flow cytometry. Graphs show data summary as mean ± SEM, pooled from 2 independent experiments. (D) NSG mice irradiated with 250 cGy at day -1 followed by injection of 3x10^6^ human pan T cells at day 0, DZNep was injected every other day intra-peritoneal (i.p.) starting at day 3 for a total of 8 doses, showing the survival graph for 10 to 20 mice in each group pooled from 4 independent experiments. (E) In vivo evaluation of DZNep toxicity, NSG mice irradiated with 250 cGy followed by DZNep injection at day 4 post irradiation every other day for total of 8 i.p. injections, 3–4 mice in each treatment group. Unpaired t-test was used to compare between groups in A and B. Log-rank test used to compare between curves in C, D and E. * p< 0.05, ** p< 0.01, *** p< 0.001, **** p< 0.0001. BM = bone marrow only without T cells infusion group, irr = irradiation only without T cells infusion group.

Pre-treatment of murine pan T cells with DZNep (0.2 μM) showed a moderate inhibition of EZH2 expression at day 3 post-activation with anti-CD3/CD28 antibody-coated beads (Fig 1A). However, there was significant apoptosis in DZNep (0.2 μM) treated T cells in comparison to the DMSO control as measured by a 4-7-fold increase in Annexin V staining, for both CD4 and CD8 subsets, respectively (p < 0.0001 for both) (Fig 1A). To determine if DZNep can prevent GvHD in our fully MHC mismatched mouse model of GvHD (B6 → Balb/c), we treated mice with DZNep (1mg/kg) every other day, post-transplant, starting on day 3 for total of 8 doses. Mice treated with DZNep had reduced GvHD as determined by significant improvement in survival compared to mice treated with vehicle control (survival at day 60 post HCT, DZNep 70% Vs. DMSO 6.6% p = 0.0002 Fig 1B). These data confirm the findings of He et al, demonstrating that DNZep reduces GvHD in murine models of allo-HCT[11].

### Effect of DZNep in human T cells

To determine if DZNep effectively inhibited EZH2 expression in human T cells we purified human pan T cells and treated them with DZNep prior to activation with anti-CD3/CD28 antibody-coated beads. DZNep was significantly less effective at inhibiting EZH2 expression in human CD4 T cell compared to murine CD4 T cells and required 5-fold increase in DZNep concentration to achieve at least 40% inhibition (Fig 1C). There was a small but significant increase in T cell apoptosis in human T cells treated with DZNep (0.2 μM) relative to DMSO in both CD4 (1.8-fold p = 0.021) and CD8 (1.8-fold p < 0.0001) human T cell subsets (Fig 1C). This indicates that DNZep was less effective at inducing apoptosis in human T cell compared to murine T cell, which demonstrated a 4-fold increase in Annexin V staining for CD4 and 7-fold increase for CD8 subsets (Fig 1A). Of note, even though DZNep significantly inhibits EZH2 expression in human CD8 T cells, suppression of EZH2 did not result in robust CD8 T cell apoptosis (Fig 1C). In addition, a five-fold increase in DZNep concentration (compared to what is seen in mouse T cells) was necessary to only modestly decrease EZH2 expression in both human CD4 and CD8 T cells but without any further increase in cell apoptosis compared to the concentration of 0.2 μM. Overall, these data suggest that human T cells are less sensitive to DZNep-induced cell death compared to murine T cells. These data further suggest that DZNep might be less effective at reducing GvHD induced by human T cells in vivo. To assess the efficacy of DZNep in human T cell induced xenogeneic GvHD, we used a human T cell xenograft mouse model of GvHD. Sub-lethally irradiated NSG mice (250 cGy) were infused with human panT cells (3 x 10^6^) via the lateral tail vein on day 0. Mice were treated with DZNep (doses 0.2, 1, or 5 mg/kg) or vehicle. In contrast to the GvHD mitigating effect of DZNep in murine models of GvHD, DZNep accelerated death of xenograft recipients compared to untreated controls (Fig 1D). To test whether this detrimental effect is related to the drug toxicity or accelerated GvHD, we sub-lethally irradiated NSG mice and treated them with various dose levels of DZNep or vehicle without transferring human T cells (Fig 1E). At day 20 after irradiation all mice treated at higher DZNep doses died, likely from DZNep toxicity, while mice treated with vehicle or DZNep (0.2 mg/kg) did not die. In addition, even though NSG mice without human T cell transfer were able to tolerate the lower dose of DZNep (0.2 mg/kg), this dose could not prevent or mitigate GvHD in xenograft recipient mice (survival at day 40 post human T cells transfer, DZNep 0% vs. DMSO 25% p = 0.0464) (Fig 1D).

### Selective pharmacological inhibition of H3K27me3 with EPZ6438 fails to prevent GvHD

**Fig 2:**
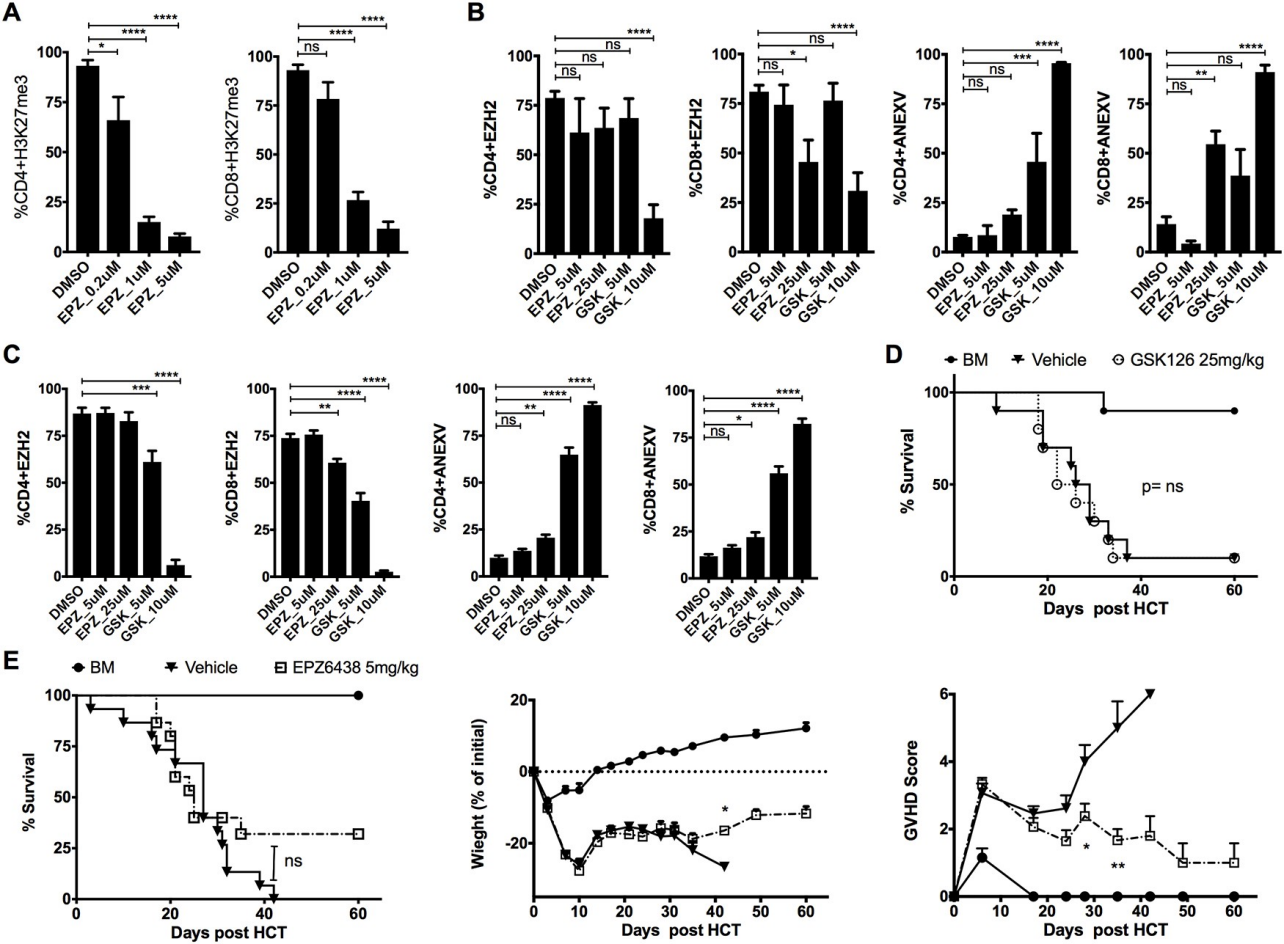
Selective inhibition of H3K27me3 is not effective in GvHD prevention. (A, B) Pan T cells isolated form B6 mouse spleen and treated at day 0 with EPZ6438, GSK126 or 10% DMSO followed by activation with anti-CD3/CD28 antibody-coated beads for 3 days. T cells were then harvested and analyzed by intracellular staining for H3K27me3 and EZH2 or staining using Annexin V detection kit by flow cytometry. Graphs show data summary as mean ± SEM, pooled from 4 independent experiments. (C) Human pan T cells isolated from PBMCs and treated at day 0 with EPZ6438, GSK126 or 10% DMSO followed by activation with anti-CD3/CD28 antibody-coated beads for 3 days, T cells then harvested and followed by intracellular staining for EZH2 or staining using Annexin V detection kit by flow cytometry. Graphs show data summary as mean ± SEM, pooled from 2 independent experiments. (D) MHC mismatched HCT (B6 → Balb/c) was performed as described in methods. Vehicle (10%DMSO) or GSK126 was injected every other day at 25 mg/kg i.p. starting at day 3 post-transplant for a total of 8 doses, showing the survival graph with 10 mice in each group pooled from 2 independent experiments. (E) MHC mismatched HCT (B6 → Balb/c) was performed as described in methods, vehicle (10% DMSO) or EPZ6438 was injected daily at 5 mg/kg s.c. starting at day 3 post-transplant for total of 15 doses, showing the survival graph, weight chart, and GvHD clinical score with 15 mice in each treatment group pooled from 3 independent experiments. One-way ANOVA was used to compare between groups in A, B and C. Log-rank test used to compare between survival curves in D and E. Multiple t-tests were used to compare between groups at multiple time points in weight chart and GvHD clinical score graphs. * p< 0.05, ** p< 0.01, *** p< 0.001, **** p< 0.0001. BM = bone marrow only group.

DZNep globally inhibits activating and repressing histone methylation marks raising a concern about the broad and off target effects[15]. For example, H3K4me3 is activating histone methylation mark that is enriched in pathways of T cell receptor signaling, Janus kinase (JAK)-STAT signaling and several molecules involved in cell-cycle progression [16]. We tested a selective small molecule inhibitor of H3K27me3, EPZ6438, and found that EPZ6438 effectively inhibits H3K27me3 in activated murine T cells in vitro (Fig 2A). However, EPZ6438 fails to effectively inhibit EZH2 expression in CD4+ T cell at all concentrations tested and only weakly inhibited EZH2 expression in CD8 T cells at high concentration (25 μM) (Fig 2B). We next tested the ability of GSK126, another selective small molecule inhibitor of H3K27me3, to inhibit EZH2 and found no significant inhibition on the expression of EZH2 by GSK126 in murine CD4 or CD8 subsets at the lower concentration (5 μM) (Fig 2B). Nonetheless, there was significant cell apoptosis. Both CD4 and CD8 T cell subsets showed that apoptosis is significantly associated with EZH2 inhibition at the higher GSK126 concentration (Fig 2B). Collectively, selective inhibition of H3K27me3 using EPZ6438 and GSK126 exhibits associated EZH2 inhibition at high concentrations mainly in CD8 subsets. A similar observation was also seen with activated human pan T cells treated with EPZ6438 and GSK126 (Fig 2C). To determine the ability of selective pharmacological inhibition of H3K27me3 to prevent GvHD, we used a fully MHC mismatched murine GvHD model (B6→Balb/c allo-HCT) and treated mice with EPZ6438, GSK126 or vehicle. We found no significant improvement in mouse survival, regardless of dose, comparing with the vehicle control group even though mice treated with EPZ6438 at 5 mg/kg showed marginal improvement in body weight and clinical GvHD score compared to the vehicle control group (Fig 2D and 2E) and (S1 Fig). Overall, we conclude that pharmacologic inhibition of H3K27me3 does not protect the allo-HCT murine recipients from GvHD. Drug toxicity might be an explanation for the inability of EPZ6438 to show any survival advantage although we found no significant adverse effect of EPZ6438 and GSK126 on bone marrow engraftment and hematopoiesis (S2 Fig).

## Discussion

We sought to determine if pharmacologic inhibition of EZH2 was effective at mitigating GvHD mediated by human T cells. In contrast to the robust GvHD protection of DZNep in mouse model of GvHD, DZNep was associated with early death in xenogeneic GvHD model. This could be due to either acceleration of GvHD or increased toxicities. This conclusion comes from the fact that human T cells activated in vitro required a 5-fold higher concentration of DZNep to induce apoptosis compared to murine T cells, which is hypothesized to be the primary mechanism of allo-tolerance[11]. In our xenogeneic GvHD model mice treated with the higher doses of DZNep died, probably due to DZNep-related toxicity, since irradiated NSG mice without human T cell transfer died at approximately the same time after treatment when treated with the same high dose of DZNep, including 1mg/kg dose. We speculated that the increased sensitivity toward any potential toxic effect of DZNep treatment was likely secondary to the defective innate immunity and impaired DNA repair of this specific mouse model especially after irradiation exposure[17]. We also speculated that a lower tolerable dose of DZNep was not sufficient to induce T cell apoptosis but was enough to enhance T cell differentiation that resulted in more severe GvHD, as previously reported with ex vivo DZNep treatment of murine T cell [18]. Furthermore, there are fundamental differences between fully murine models of GvHD and xenogeneic models of GvHD. In the latter, human T cell xeno-reactivity depends on xenoantigens that are usually associated with a more robust immune response than alloantigens, which might not be affected by lower doses of DZNep[19]. In addition, it is also possible that DZNep-mediated EZH2 inhibition might result in paradoxical effects by enhancing T cell differentiation through removing the repressive histone methylation marks from effector T cell loci such as *Ifng*, as previously described[18, 20]. We found no strong evidence to support that selective pharmacologic inhibition of H3K27me3 with EPZ6438 or GSK126 could reduce GvHD in a fully MHC mismatched mouse allo-HCT model. These data suggest that the effect of EZH2 (or PRC2) on GvHD pathogenesis is not dependent on the selective tri-methylation of H3K27, as previously described [10]. Although EPZ6438 was the least potent agent to induce T cell apoptosis in vitro, there was some improvement in GvHD severity for which one might speculate another potential mechanism for the control of alloreactive T cells, such as regulatory T cells. However, we did not evaluate this part mainly because EZH2 is essential for regulatory T cells induction and maintenance, for that we expect regulatory T cells to be defective after the use of EZH2 or H3K27m3 small molecule inhibitors as previously reported [9, 11, 21]. Epigenetic mapping showed that T cell differentiation and plasticity is dependent on critical balance between activating and repressing histone methylation marks [18, 22]. These reports suggest that specific methylation of histones might not be an effective strategy to modulate the allo-reactivity of donor T cells since we show here that pharmacologic inhibition of H3K27me3 with GSK126 and EPZ6438 had no effect on GvHD in a major MHC mismatched murine allo-HCT model. To add additional complexity, *Ezh2* genetic deficiency or in vitro treatment of donor T cells with DZNep has recently been found to be associated with increased sensitivity of T cells to cytokine polarization. Furthermore, *Ezh2* deficiency in vivo was found to worsen mouse asthma allergy and associated with accumulation of Th2 cytokine-producing cells[18]. While histone modification of the T cell lineage-specific transcription factors could play a role in transplant tolerance, it remains unclear whether activating and/or repressing histone methylation marks play a role in post-transplant immune tolerance. Huang et al. have recently reported the role of EZH2 destabilization through heat shock protein (Hsp) 90 inhibition, independent of histone methylation in various GvHD mouse models as a pathway for reducing GvHD [23], further questioning the role of EZH2/PRC2-mediated histone methylation in alloreactivity.

In summary, we showed that DZNep was not effective in preventing GvHD in a xenogeneic HCT model. Further work to determine the exact mechanism of high dose DZNep-associated early death in xenogeneic HCT model is needed. We also show that selective inhibition of H3K27me3, using two small molecule inhibitors, was not sufficient to prevent GvHD using a major MHC mismatched murine allo-HCT model. Novel interventions to directly inhibit EZH2 (the gene or protein) or indirectly through other components of the PRC2 may be a more rational approach to induce transplant tolerance and reduce GvHD in vivo. It is possible that gene editing of EZH2 using CRISPR/Cas9 or other similar approaches in human T cells may also provide a viable and more effective alternative to inhibit the lysine N-methyltransferase enzymatic activity of EZH2/PRC2 which by itself is not sufficient to limit alloreactivity.

## Supporting information

S1 Fig Multiple dose level for GSK126 and EPZ6438 used to treat mice after allo-HCT. MHC mismatched HCT (B6 → Balb/c) was performed as described in methods. (A) Vehicle (10%DMSO) or GSK126 was injected every other day at 100 mg/kg, 50 mg/kg, or 25 mg/kg i.p. starting at day 3 post-transplant for a total of 8 doses, showing the survival graph with 10 mice in each group pooled from 2 independent experiments. (B) Vehicle (10% DMSO) or EPZ6438 was injected daily at 100 mg/kg, 50 mg/kg, or 25 mg/kg s.c. starting at day 3 post-transplant for total of 15 doses, showing the survival graph with 10 mice in each treatment group pooled from 2 independent experiments.
(TIFF)

S2 Fig EPZ6438 and GSK126 did not affect donor cells engraftment after murine allo-HCT. MHC mismatched HCT (B6 → Balb/c) was performed as described in methods, vehicle (10% DMSO), GSK126, or EPZ6438 was injected as described in Fig 2 legend, mice were bled at day 27 +/- 2 days. (A) Complete blood count analysis using Hemevyte machine, white blood count (WBC), hemoglobin (Hb) and platelet (PLT) (data shown for EPZ6438 only). (B) Mice whole blood was lysed then stained for CD45.2 and H2Kd for chimerism examination using flowcytometer.
(TIFF)
