# Supplementary figures and images for: Selective targeting of histone modification fails to prevent graft versus host disease after hematopoietic cell transplantation
Source: PLoS One. 2018 Nov 19;13(11):e0207609. doi: 10.1371/journal.pone.0207609 (PMC6242356; doi:10.1371/journal.pone.0207609)

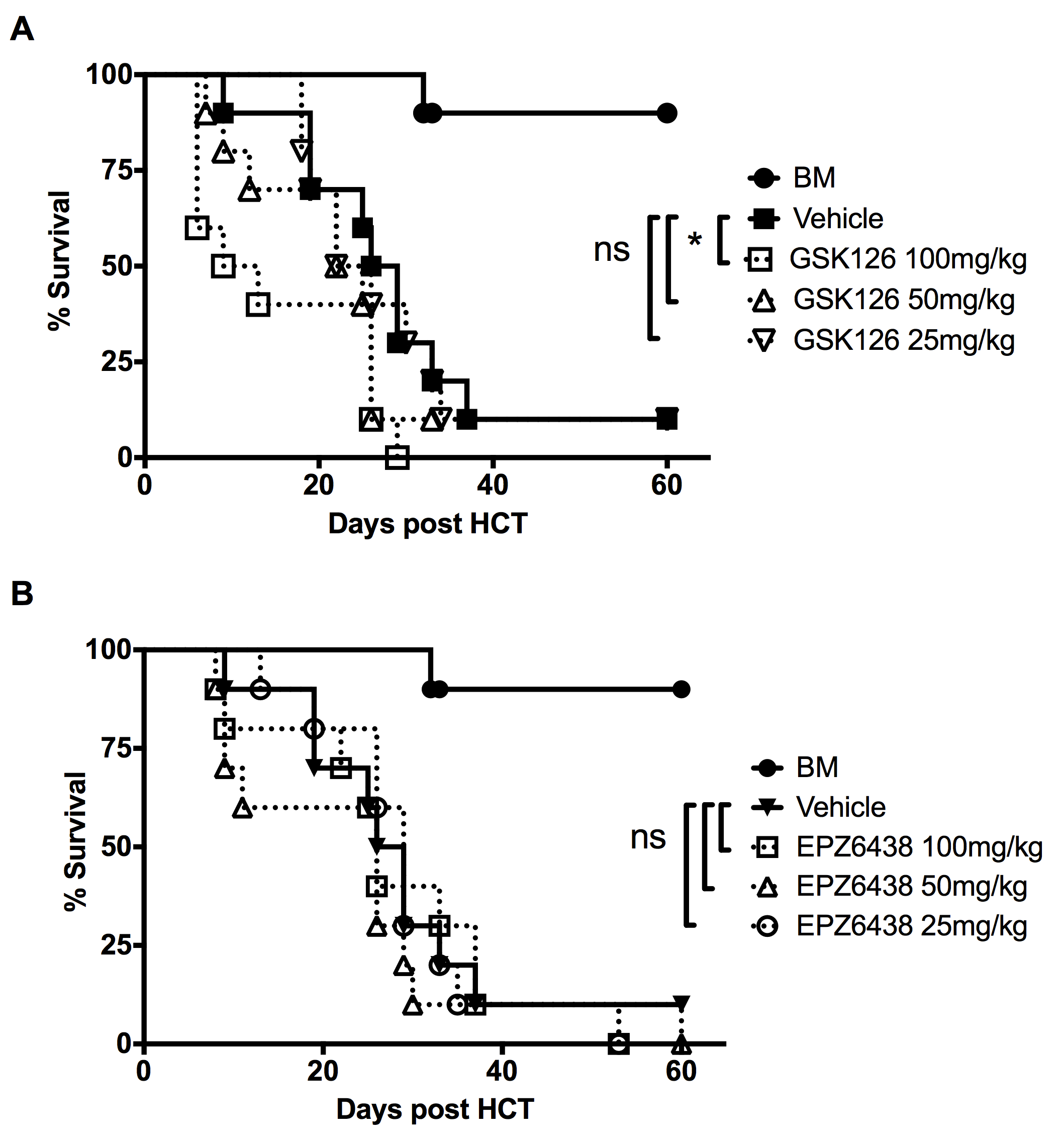

Supplement: S1 Fig — MHC mismatched HCT (B6 → Balb/c) was performed as described in methods. (A) Vehicle (10%DMSO) or GSK126 was injected every other day at 100 mg/kg, 50 mg/kg, or 25 mg/kg i.p. starting at day 3 post-transplant for a total of 8 doses, showing the survival graph with 10 mice in each group pooled from 2 independent experiments. (B) Vehicle (10% DMSO) or EPZ6438 was injected daily at 100 mg/kg, 50 mg/kg, or 25 mg/kg s.c. starting at day 3 post-transplant for total of 15 doses, showing the survival graph with 10 mice in each treatment group pooled from 2 independent experiments. (TIFF) [file pone.0207609.s001.tiff]

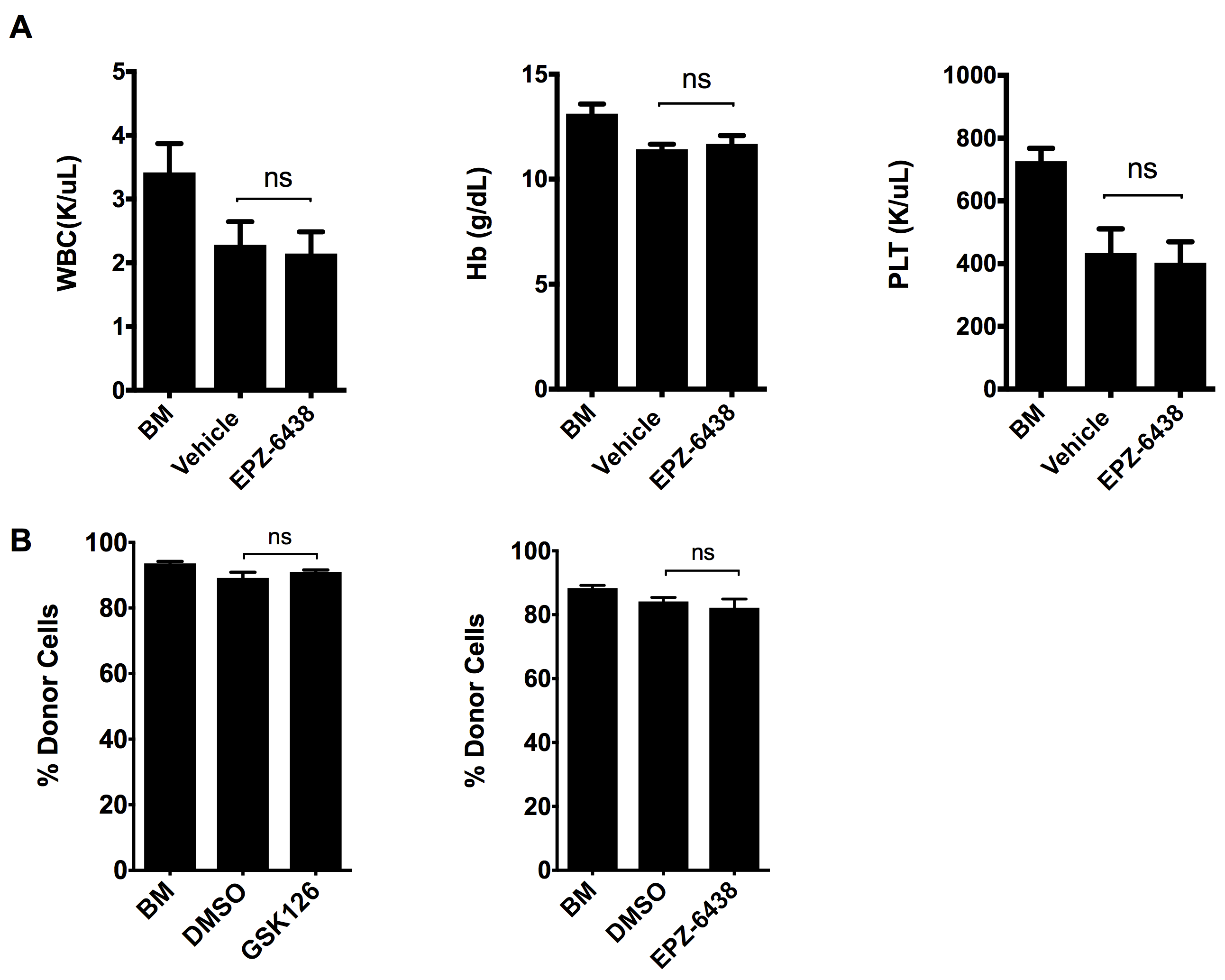

Supplement: S2 Fig — MHC mismatched HCT (B6 → Balb/c) was performed as described in methods, vehicle (10% DMSO), GSK126, or EPZ6438 was injected as described in Fig 2 legend, mice were bled at day 27 +/- 2 days. (A) Complete blood count analysis using Hemevyte machine, white blood count (WBC), hemoglobin (Hb) and platelet (PLT) (data shown for EPZ6438 only). (B) Mice whole blood was lysed then stained for CD45.2 and H2Kd for chimerism examination using flowcytometer. (TIFF) [file pone.0207609.s002.tiff]
